# Effect of baseline cognitive impairment on association between predicted propofol effect site concentration and Bispectral index or sedation score

**DOI:** 10.1186/s12871-020-01043-5

**Published:** 2020-05-28

**Authors:** Frederick Sieber, Karin Neufeld, Esther S. Oh, Allan Gottschalk, Nae-Yuh Wang

**Affiliations:** 1 Department of Anesthesiology and Critical Care Medicine, Johns Hopkins Bayview Medical Center, 4940 Eastern Avenue, Baltimore, MD 21224 USA; 2 Department of Psychiatry and Behavioral Sciences, Johns Hopkins University School of Medicine, A4Center Suite 457, 4940 Eastern Ave, Baltimore, USA; 3 Division of Geriatric Medicine and Gerontology, Psychiatry and Behavioral Sciences & Neuropathology, Johns Hopkins University School of Medicine, Mason F. Lord Building, Center Tower, 5200 Eastern Avenue, 7th Floor, Baltimore, MD 21224 USA; 4 Departments of Anesthesiology and Critical Care Medicine and Neurosurgery, Johns Hopkins Hospital, 1800 Orleans St, Baltimore, MD 21287 USA; 5 Medicine, Biostatistics and Epidemiology, The Johns Hopkins University, 2024 E. Monument Street, Suite 2-500, Baltimore, MD 21287 USA

**Keywords:** Propofol, Deep sedation, Conscious sedation, Cognitive dysfunction, Hip fractures, Bispectral index, observer’s assessment of alertness and sedation (OAA/S), Geriatric anesthesia

## Abstract

**Background:**

This study determined whether the relationship between predicted propofol effect site concentration (Ce) and observer’s assessment of alertness/sedation scale (OAA/S) or Bispectral Index (BIS) was similar comparing cognitively intact vs impaired patients undergoing hip fracture repair with spinal anesthesia and sedation.

**Methods:**

Following informed consent baseline mini-mental status exam (MMSE), Clinical Dementia Rating (CDR) and geriatric depression scale (GDS) were obtained. Intraoperatively OAA/S, BIS, and propofol (timing and exact amounts) administered were recorded. Cerebrospinal fluid was collected for Alzheimer’s (AD) biomarkers. Mean Ce level (AvgCe) during surgery was calculated using the area under the Ce measurement series from incision to closure, divided by surgical time. Average OAA/S (AvgOAA/S), and BIS (AvgBIS) were similarly calculated. Pearson correlations of AvgCe with AvgOAA/S and AvgBIS were calculated overall and by CDR. Nonparametric locally weighted scatterplot smoothing (LOWESS) fits of AvgOAA/S and AvgBIS on AvgCe were produced, stratified by CDR. Multivariable regression incorporating baseline cognitive measurements or AD biomarkers assessed AvgOAA/S or AvgBIS associations with AvgCe.

**Results:**

In 186 participants AvgBIS and AvgOAA/S correlated with AvgCe (Pearson ρ = − 0.72; *p* < 0.0001 and Pearson ρ = − 0.81; *p* < 0.0001, respectively), and remained unchanged across CDR levels. Association patterns of AvgOAA/S or AvgBIS on AvgCe guided by LOWESS fits and modeled through regression, were similar when stratified by CDR (*p* = 0.16). Multivariable modeling found no independent effect on AvgBIS or AvgOAA/S by MMSE, CDR, GDS, or AD biomarkers after accounting for AvgCe.

**Conclusions:**

When administering sedation in conjunction with spinal anesthesia, cognitive impairment does not affect the relationship between predicted propofol AvgCe and AvgOAA/S or AvgBIS.

## Background

As the population ages, the prevalence of dementia is increasing. Alzheimer’s disease (AD), the most common form of dementia in the United States (U.S.), is estimated to affect 5.7 million individuals in 2018, and the annual incidence is expected to double by 2050 [1]. Nearly one third of all people 85 years and older have AD, and it is thought that a large fraction of those that remain undiagnosed possess varying degrees of subclinical AD pathology [2]. In adults≥65 years of age undergoing surgery, preoperative cognitive dysfunction, including dementia, is common [3–5]. For instance, in the hip fracture population, dementia prevalence has been reported to be 33% [6]. Despite the increasing numbers of patients with cognitive impairment undergoing surgery, the literature provides little guidance concerning their anesthetic management. Specifically, it is unclear whether during maintenance of sedation cognitive impairment alters the relationship between predicted effect site concentration and clinically observed Bispectral Index number or sedation score.

Previous investigations examining maintenance anesthetic requirements studied general anesthesia and report no difference in sensitivity to inhalational agents [7] or infusion rate requirements with total intravenous anesthesia [8] comparing patients with and without cognitive dysfunction. While general anesthesia is certainly important to study, the number of procedures utilizing propofol as the primary means of providing procedural sedation or sedation in conjunction with regional anesthesia is enormous. However, there is little information concerning clinical anesthetic dosing requirements in patients with cognitive dysfunction during propofol sedation. We therefore performed a secondary analysis of the STRIDE (A Strategy to Reduce the Incidence of Postoperative Delirium in Elderly Patients) trial data to test the hypothesis that the clinical anesthetic dosing requirements during propofol sedation are similar comparing patients with and without cognitive impairment. The aim of this study was to determine if the relationship between predicted propofol effect site concentration (Ce) and the modified observer’s assessment of alertness/sedation scale (OAA/S) [9] or Bispectral index ((BIS) Brain Monitoring System, http://www.medtronic.com/covidien/products/brainmonitoring) number was similar during maintenance of sedation comparing cognitively intact versus cognitively impaired participants.

## Methods

### Study design and participants

The STRIDE trial was a randomized, two-group, parallel, superiority trial whose principal objective was to assess the effectiveness of lighter versus heavier sedation during spinal anesthesia in elderly patients undergoing hip fracture repair. The trial was first registered at ClinicalTrials.gov under registration number NCT00590707 on 1/2008. Johns Hopkins IRB approval was obtained for the prospective STRIDE trial on 9/27/2010 (NA_00041873). All participants provided their written informed consent.

The primary outcome of the STRIDE trial was the impact of the intervention on the incidence of delirium during postoperative Day 1 to Day 5 or to hospital discharge, whichever occurs first. These results have been previously reported [10, 11]. In short, no overall difference in the incidence of postoperative in-hospital delirium was found between the intervention groups, but a significant effect modification by level of comorbidity was observed, where lighter sedation imparts lower in-hospital delirium risk in patients with low pre-operative comorbidity [10]. In addition, when comparing lighter versus heavier sedation, there is no difference in mortality or functional outcomes of elderly hip fracture patients 1 year after surgery [11]. STRIDE was conducted at a single clinical center. A detailed description of the entire trial protocol was published previously in the supplemental material of Li et al. [12].

Briefly, patients ≥65 years old who were undergoing hip fracture repair with spinal anesthesia and propofol sedation and who did not have preoperative delirium or severe dementia were randomized to receive either heavier (OAA/S 0–2) or lighter (OAA/S 3–5) intraoperative sedation. The inclusion criteria were 1) admission to Johns Hopkins Bayview Medical Center for surgical repair of traumatic hip fracture; 2) 65 years of age or older; 3) a preoperative mini-mental status exam (MMSE) [13] score of 15 or higher; and 4) receiving spinal anesthesia. The exclusion criteria included 1) receiving general anesthesia; 2) inability to speak or understand English; 3) severe chronic obstructive pulmonary disease or congestive heart failure; 4) refusal to give informed consent; 5) non-participating attending surgeon; 6) hip fractures in both hips on same admission; 7) repair of another fracture concurrently with the hip fracture; 8) prior hip surgery on the same hip to be repaired in the current surgery; and 9) preoperative delirium.

### Data collection at baseline prior to surgery

Prior to surgery, baseline MMSE, modified Clinical Dementia Rating (CDR) as previously described [14], geriatric depression scale (GDS) [15], and Charlson comorbidity index (CCI) [16] were obtained, in addition to demographic information. CDR was adjudicated by a consensus diagnosis panel [10]. CDR scores were classified as follows: 0 = normal, 0.5 = mild cognitive impairment, ≥1 = dementia. Evaluations using Confusion Assessment Method (CAM) [17], Delirium Rating Scale-R-98 (DRS-R-98) [18], and abbreviated digit span test (DST) were also collected at baseline and used to confirm absence of preoperative delirium.

### Intervention

After satisfactory administration of spinal anesthesia, the patient was randomly assigned to one of two groups in blocks with equal allocation, stratified by age and MMSE at baseline. Intra-operatively, one group had the depth of sedation maintained at an OAA/S score of 0–2. This was the heavier sedation group. Patients in the other group had the depth of sedation maintained at an OAA/S score of 3–5. This was the lighter sedation group.

### Data collection during surgery

The propofol was titrated individually for each participant to achieve and maintain the depth of sedation required by that participant’s assigned treatment group (lighter or heavier sedation). The depth of sedation for all participants was measured by the OAA/S, administered every 15 min intra-operatively. During the intraoperative study period, the BIS was also recorded. The BIS monitor readout was covered throughout the surgery so that the Study Anesthesiologist/Anesthetist remained masked to the BIS values while administering propofol. The BIS readings served as an independent measure of the level of adherence to the trial interventions. Mean arterial blood pressure (MAP) was measured via oscillotonometry every 5 min, and automatically recorded in the electronic medical record. After surgery, the MAP values were abstracted and entered into the database. After surgery, the BIS values were abstracted, matched in time to their corresponding MAP values, and entered into the database at 5 min intervals. During surgery propofol was administered intravenously using the Alaris PC 8100 series infusion pump which gives continuous output of total volume (ml) infused. A continuous download of total propofol volume infused was obtained for each study case. From this data, the predicted propofol Ce was calculated on a minute by minute basis using the method of Schnider et al. [19]. Ce values were then matched in time to their corresponding MAP values and entered into the database at 5-min intervals.

Mean predicted Ce level (AvgCe) during surgery for each participant was calculated based on the area under the Ce measurement series from incision to end of surgery, divided by the surgery time --- the length of time between incision and end of surgery. Average OAA/S (AvgOAA/S), BIS (AvgBIS) and MAP (AvgMAP) levels during surgery were calculated using the same approach. The Ce values matched in time to the corresponding MAP values were used in the calculation of avgCe. Similarly, the BIS and OAA/S values matched in time to the corresponding MAP values were used in the calculation of avgBIS and avgOAA/S, respectively.

Prior to the administration of spinal anesthesia, approximately 6 cc of cerebrospinal fluid (CSF) was collected and stored for later analysis of AD biomarkers [14].

### Statistical analysis

Distributions of baseline characteristics before surgery and measurements during surgery were described. Mean and standard deviations (SDs) were calculated for continuous variables and frequency distributions (n and %) were reported for categorical variables. Potential level of systematic bias in using AvgOAA/S and AvgBIS as proxy measures of AvgCe during surgery were assessed using Bland-Altman (B-A) plots. Given that AvgOAA/S and AvgBIS were both significantly correlated with AvgCe with negative correlations, reversed variables (5-AvgOAA/S and 100-AvgBIS, respectively) were used in the B-A analysis. Due to the differences in range of score for these 3 variables, all three variables were also standardized to have mean = 0 and SD = 1 to produce the B-A plots. Pearson correlations were calculated overall and by CDR and CCI levels. To explore whether the associations between AvgOAA/S or AvgBIS and AvgCe were influenced by cognitive measurement or comorbidity, nonparametric locally weighted scatterplot smoothing (LOWESS) fits of AvgOAA/S and AvgBIS on AvgCe were produced, stratified by the CDR score levels (0, 0.5, and ≥ 1). Similar LOWESS fits were also derived according to the CCI levels (0, 1, 2, and > 2). For ease of interpretation, nonlinear associations suggested by the LOWESS fit were approximated by linear or segmental linear models, as appropriate, in subsequent regression analyses. To better understand the relationship between AvgOAA/S or AvgBIS and AvgCe, multivariable regression analyses incorporating baseline cognitive measurements or CSF biomarkers as potential effect modifiers were performed to assess potential differentiation of associations of AvgOAA/S and AvgBIS with AvgCe by levels of these variables. Analyses were carried out using SAS 9.4, and an estimated association or interaction with *p*-value < 0.05 was considered statistically significant.

## Results

Table 1 shows the baseline characteristics for this patient group. Exact timing of propofol change in infusion rates and boluses was recorded in 186/200 participants. Thus, we focused the analyses on these 186 participants. BIS data was not recorded in *n* = 3 and baseline CDR was not completed in *n* = 2 patients. As an ancillary study to the main STRIDE trial, cerebrospinal fluid biomarkers were obtained in *n* = 178 patients. No statistically significant difference in baseline characteristics between these 186 participants and those 14 patients who did not have predicted Ce measurement was noted (Table 1).

**Table 1:** Patient characteristics in Mean (SD) or n (%) at baseline and measurements during surgery

Baseline characteristics	With Ce (*n* = 186)	Without Ce (*n* = 14)
**Before surgery**		
Age	81.9 (7.8)	79.8 (7.0)
Mini-Mental Status Examination (MMSE)	24.2 (3.7)	25.1 (3.4)
Geriatric depression scale score	3.9 (3.6)	3.0 (2.0)
Clinical Dementia Rating (*n* = 184/14)		
0	75 (41%)	7 (50%)
0.5	88 (48%)	6 (43%)
≥ 1.0	21 (11%)	1 (7%)
Charlson Comorbidity Score	1.5 (1.7)	2.0 (2.4)
0	66 (35%)	6 (43%)
1	83 (45%)	3 (21%)
2	26 (14%)	3 (21%)
> 2	11 (6%)	2 (14%)
**Alzheimer’s cerebrospinal fluid biomarkers**		
Total Tau, pg/ml (*n* = 164/6)	490.8 (275.1)	617.7 (435.7)
Phosphorylated Tau (pg/ml (*n* = 163/6)	56.7 (25.1)	63.0 (32.9)
Amyloid Beta 42 (pg/ml (n = 164/6)	298.3 (164.1)	281.2 (106.7)
Amyloid Beta 42/Total Tau(n = 164/6)	0.71 (0.38)	0.61 (0.32)
Amyloid Beta 42/Phosphorylated Tau (n = 163/6)	5.75 (2.83)	5.16 (2.29)
**During surgery**		
Observers Assessment Alertness/Sedation score	2.2 (2.1)	1.4 (2.1)
Bispectral Index number (*n* = 183/13)	69.1 (18.1)	75.0 (13.7)
Brain Effect Site Concentration, ug/ml	1.26 (0.79)	--- (−--)
Mean Arterial Pressure	73.4 (10.5)	77.1 (11.8)

CDR, Clinical Dementia Rating (0 = normal, 0.5 = mild cognitive impairment, ≥1 = dementia); MMSE, Mini-Mental Status Examination (maximum score of 30 points)

### Systematic bias of OAA/S and BIS measurements against Ce

**Fig. 1:**
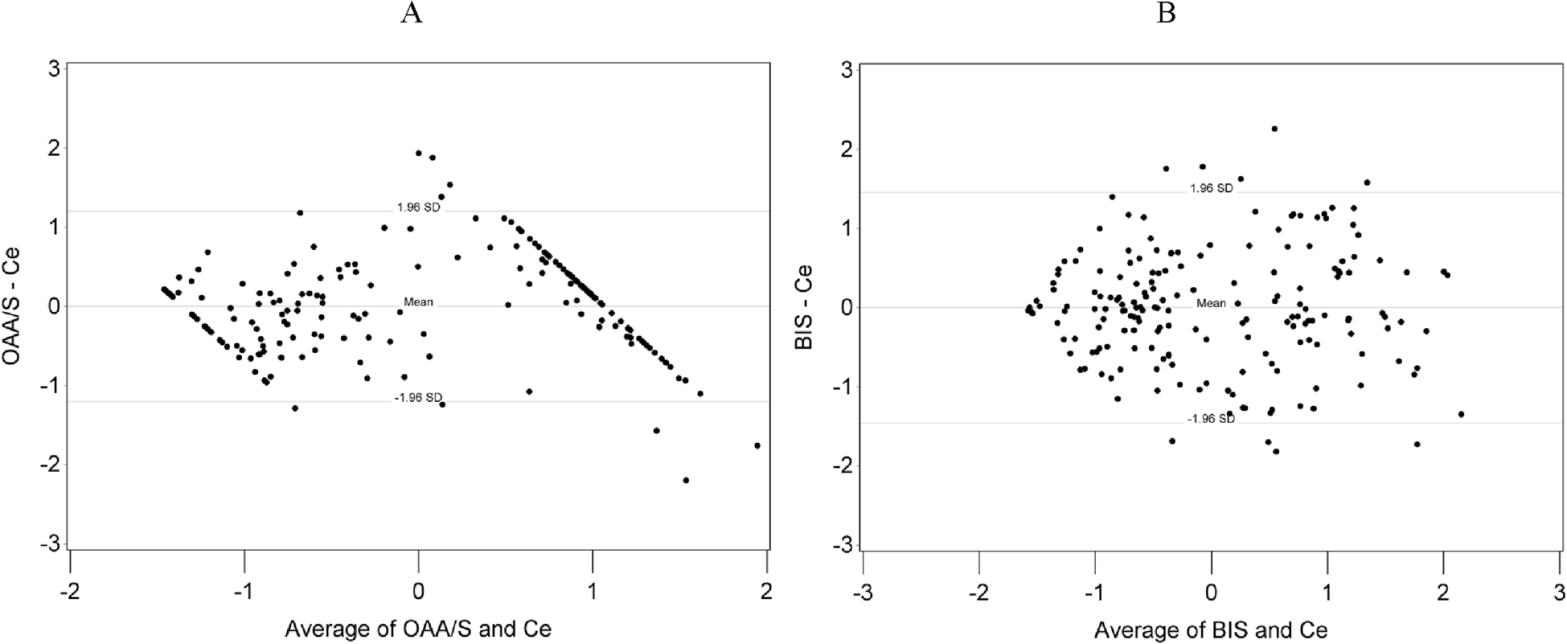
Bland-Altman plots. **a** shows Bland-Altman plot for OAA/S and Ce. Y-axis is calculated difference between OAA/S and Ce; X-axis is mean of OAA/S and Ce. OAA/S values were reversed in order (i.e., 5 – OAA/S was used). OAA/S and Ce values were then standardized to have respective distributions both with mean of 0 and standard deviation of 1. **b** shows Bland-Altman plot for BIS number and Ce. Y-axis is calculated difference between BIS number and Ce; X-axis is mean of BIS number and Ce. BIS values were reversed in order (i.e., 100 – BIS was used). BIS and Ce values were then standardized to have respective distributions both with mean of 0 and standard deviation of 1

Over the observed range of AvgOAA/S or AvgBIS, no systematic bias in using them as proxy measures of AvgCe during surgery was found (Fig. 1). The mean difference of AvgOAA/S and AvgBIS from AvgCe were both 0, with 95.2% of the observed differences within the range of +/− 1.96SD.

### BIS determinants

AvgBIS was strongly correlated with AvgCe during surgery (Pearson ρ = − 0.72; *p* < 0.0001), and the correlation remained strong across CDR levels (Table 2).

**Table 2:** Correlations between AvgCe and AvgBIS or Avg OAA/S, overall and by clinical dementia rating

	Avg BIS		Avg OAA/S	
	Pearson	P	Pearson	P
Overall	−0.72	<.0001	−0.81	<.0001
Clinical Dementia Rating				
0	−0.81	<.0001	− 0.86	<.0001
0.5	−0.67	<.0001	−0.80	<.0001
≥ 1	−0.58	0.0070	−0.78	<.0001

The correlation seemed the strongest in patients with CDR = 0, with a trend toward weaker correlation as cognitive decline measured by CDR worsened. The strong correlation between AvgBIS and AvgCe was also maintained throughout the range of preoperative Charlson co-morbidity scores observed in the STRIDE population (Table 3).

**Table 3:** Correlations between AvgCe and AvgBIS or Avg OAA/S by level of Charlson co-morbidity scores

	Avg BIS		Avg OAA/S	
	Pearson	P	Pearson	P
Charlson Co-Morbidity Index				
0	−0.80	<.0001	−0.86	<.0001
1	−0.68	<.0001	−0.81	<.0001
2	−0.76	<.0001	−0.73	<.0001
> 2	−0.61	0.05	−0.94	<.0001

There was no statistically significant differentiation on the level of association between AvgBIS and AvgCe by CDR or CCI levels.

Linear regression modeling demonstrated that AvgBIS was strongly predicted by AvgCe (*p* < .0001) and less so by avgMAP (*p* = 0.01). AvgBIS and AvgMAP correlated at CCI = 0 (r = 0.37; *p* = 0.002). However, at higher CCI scores correlations were statistically not significant between AvgBIS and AvgMAP (Table 4).

**Table 4:** Correlations between AvgBIS and AvgMAP, overall and by Charlson score and clinical dementia rating

	Pearson	P
Overall	0.21	0.004
Charlson Co-Morbidity Index		
0	0.37	0.002
1	0.12	0.29
2	0.14	0.50
> 2	0.27	0.42
Clinical Dementia Rating		
0	0.18	0.12
0.5	0.31	0.004
≥ 1	−0.11	0.66

Modest correlations existed between AvgBIS and AvgMAP at mild cognitive impairment (MCI, CDR = 0.5) level only.

### OAA/S determinants

AvgOAA/S was strongly correlated with AvgCe during surgery (Pearson ρ = − 0.81; *p* < 0.0001), and the correlation remained strong across CDR levels (Table 2). The correlation seemed the strongest in patients with CDR = 0, with a trend toward slightly weaker correlation as cognitive decline measured by CDR worsen. The overall correlation between AvgOAA/S and AvgCe was strong and maintained throughout the range of preoperative Charlson co-morbidity scores observed in the STRIDE population (Table 3). AvgOAA/S and AvgMAP did not correlate throughout the range of preoperative CCI and CDR scores observed in the STRIDE population. Results from linear regression modeling also demonstrates that AvgOAA/S level is strongly predicted by AvgCe (*p* < .0001), but not by AvgMAP.

### Effect of baseline cognition or comorbidity

**Fig. 2:**
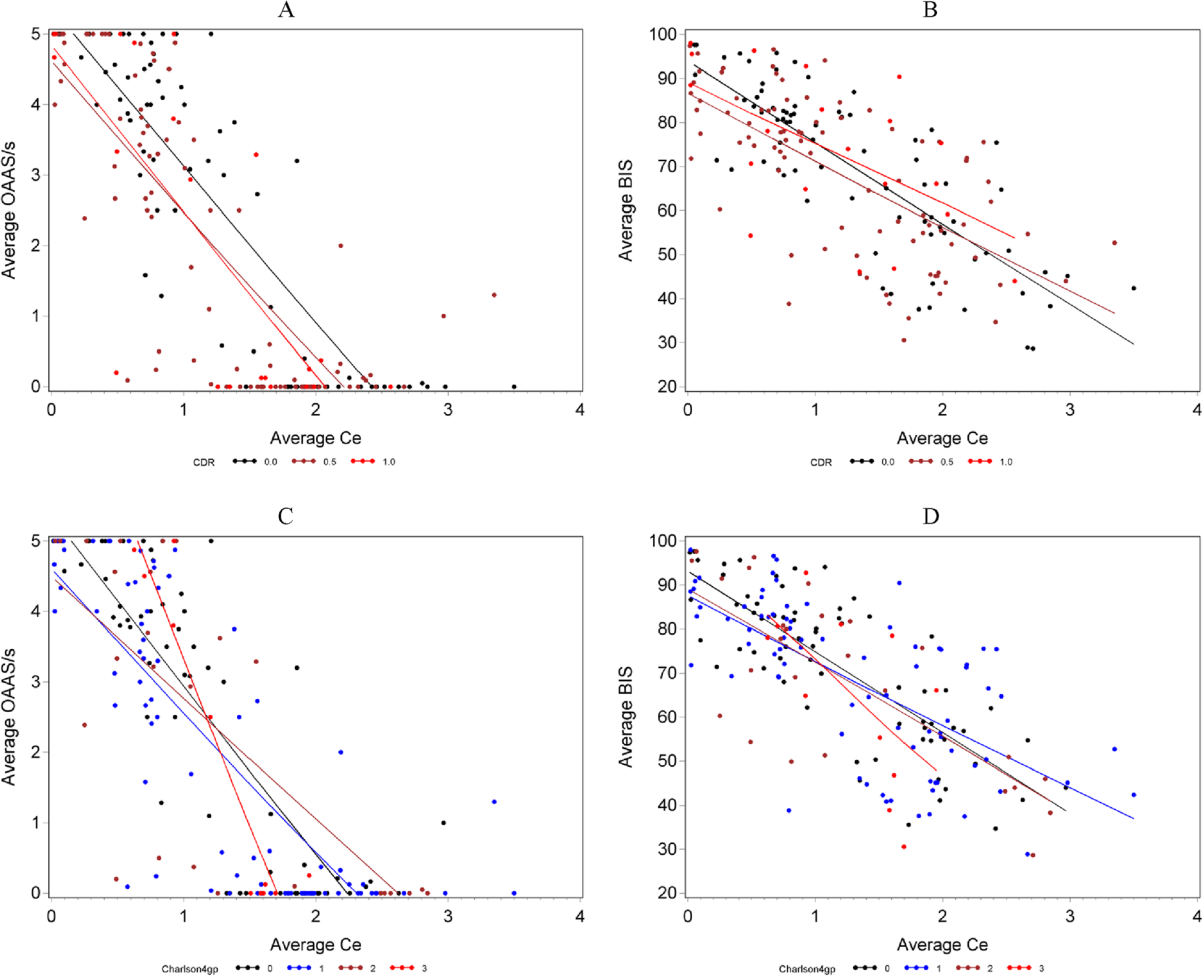
Nonparametric locally weighted scatterplot smoothing (LOWESS) fits of AvgOAA/S and AvgBIS on AvgCe stratified by the Clinical Dementia Rating (CDR) score levels and Charlson Co-morbidity Index (CCI) score levels. **a**: Nonparametric LOWESS fit of AvgOAA/S on AvgCe stratified by the CDR score levels (0, 0.5, and ≥ 1) with fitted lines for each CDR score level. **b**: Nonparametric LOWESS fit of AvgBIS on AvgCe stratified by the CDR score levels (0, 0.5, and ≥ 1) with fitted lines for each CDR score level. **c**: Nonparametric LOWESS fit of AvgOAA/S on AvgCe when stratified by the CCI score levels (0, 1, 2, and ≥ 3) with fitted lines for each CCI score level. **d**: Nonparametric LOWESS fit of AvgBIS on AvgCe when stratified by the CCI score levels (0, 1, 2, and ≥ 3) with fitted lines for each CCI score level

Regression analyses based on association patterns suggested by nonparametric LOWESS fits of AvgOAA/S on AvgCe showed no statistically significant difference between association patterns when stratified by either the CDR score levels (0, 0.5, and ≥ 1; *p* = 0.16) or the CCI score levels (0, 1, 2, and ≥ 3; *p* = 0.37) (Fig. 2, A and C). Similarly, association patterns suggested by LOWESS fits of AvgBIS on AvgCe were not statistically different when stratified by either the CDR score levels (0, 0.5, and ≥ 1) or the CCI score levels (0, 1, 2, and ≥ 3) (Fig. 2, B and D). AvgOAA/S and avgBIS were highly correlated. At CDR 0, 0.5, and 1 or 2, the Pearson correlation was 0.81 (*p* < 0.0001), 0.76 (p < 0.0001), and 0.63 (*p* = 0.002), respectively. Multivariable modeling found no independent effect on AvgBIS by either MMSE, CDR, CDR sum of boxes, GDS, or any cerebrospinal fluid AD biomarker (data not shown). Other variables tested without independent effect on AvgBIS included age, BMI, and CCI. Regression modeling found no association between AvgOAA/S and either MMSE, CDR, CDR sum of boxes, or any cerebrospinal fluid AD biomarker. Two interactions independently predicted AvgOAA/S. AvgOAA/S was predicted by an age-AvgCe interaction as well as an age-GDS interaction (Table 5), where the same level of increase in AvgCe was observed with greater decrease in AvgOAA/S score in older patients than in younger patients, and that patients with more severe depressive symptoms before surgery were observed with lower AvgOAA/S score during surgery than patients with less severe depressive symptoms with greater magnitude in this sedation score differences among older patients than in younger patients. Other variables tested without independent effect on AvgOAA/S included BMI, and CCI.

**Table 5:** Multivariate Regression Analysis for Predictors of AvgOAA/S

Parameter	Estimate	Standard Error	t Value	P
Intercept	0.39	0.699	0.56	0.57
AvgCe	−1.55	0.137	−11.36	<.0001
AvgMAP	0.01	0.007	1.69	0.09
AvgBIS	0.04	0.006	7.15	<.0001
Age	0.02	0.021	0.83	0.41
AvgCe and Age Interaction	−0.03	0.011	−2.83	0.005
Geriatric Depression Score (GDS)	−0.02	0.021	−1.07	0.29
GDS and Age Interaction	−0.01	0.002	−2.34	0.020

## Discussion

This study did not find evidence to support that the associations between predicted propofol AvgCe and both AvgOAA/S and AvgBIS during sedation among elderly patients undergoing hip fracture repair were significantly altered by baseline CDR or any of the other baseline cognitive variables analyzed.

The current study reports that during sedation, the predicted propofol AvgCe and to a lesser extent AvgMAP affect AvgBIS. The literature reports that BIS, blood propofol concentration [20], Ce [21], and blood pressure are closely related [22]. However, in clinical circumstances where blood pressure decreases occur independent of anesthetic cardiovascular effects, decreases in brain perfusion may be associated with lower BIS numbers independent of anesthetic depth [23–25]. Even less dramatic changes in blood pressure have been associated with BIS modifications. For instance, during stable anesthetic maintenance, blood pressure and BIS are independently correlated (r = 0.696) during positional change from supine to beach chair in preparation for shoulder surgery [26]. The mean BIS level during sedation was not independently affected by any of the baseline cognitive measurements including CDR and CDR sum of boxes, MMSE, or CSF AD biomarker levels. Previous investigations examining the effects of cognitive dysfunction on maintenance anesthetic dosing requirements studied only general anesthesia, used multiple agents and analyzed either retrospective data [7] or lacked adequate power [8]. In addition, the severity of dementia was incompletely characterized using either baseline MMSE [8] or history of dementia to establish diagnosis [7]. These studies reported no difference in sensitivity to inhalational agents (as defined by BIS only) [7] or infusion rate requirements using the total intravenous combination of remifentanil and propofol [8] during maintenance of general anesthesia when comparing patients by presence or absence of cognitive dysfunction. Our study is unique in its focus on sedation, using more than simply BIS to assess sedation depth, and careful classification of preoperative cognitive state. In addition, the use of a single anesthetic agent is pertinent to procedural sedation which in actual practice is often accomplished with only the single agent propofol.

The current study reports that during sedation, the predicted propofol AvgCe affects AvgOAA/S in confirmation of previous reports demonstrating correlations between the blood propofol concentration and OAA/S [27]. AvgOAA/S was affected by an age-AvgCe interaction such that the same level of increase in AvgCe would observe greater decrease in AvgOAA/S score in older patients than in younger patients. This is consistent with Schnider et al. [19] who observed increasing sensitivity to propofol in elderly patients for loss of consciousness. Both age and level of spinal anesthesia have been reported to modify sedation scores [28]. In the current study, age is the more important modifying factor of OAA/S as the overall level of spinal anesthesia demonstrated little variation among participants (T9 ± 1.5 dermatomes). AvgOAA/S was also predicted by age-GDS interaction such that higher GDS scores in conjunction with age were associated with lower AvgOAA/S score at the same Ce. The GDS is a tool to assess symptoms of depression, and there is evidence to suggest that depression and cognitive impairment may be due to related brain dysfunction [29]. As the interaction between GDS and age in determining OAA/S score have not previously been reported, it will require confirmation. Similar to AvgBIS, AvgOAA/S score was not influenced by any of the baseline cognitive measures performed in the STRIDE study.

### Strengths

Exact amounts and administration time of propofol was recorded allowing for calculation of predicted Ce. Multiple assessments of baseline cognition were performed in this study and all were able to be tested to verify the robustness of our conclusions. Level of sedation was assessed both behaviorally as well as with BIS. Little systematic bias was observed for both means of assessing sedation depth defined by predicted Ce.

### Limitations

Fourteen patients were not included in analysis. However, the baseline characteristics of these 14 patients are comparable to the 186 participants included in this report, and sensitivity analysis demonstrates that the study results would not have been affected by these additional 14 patients on analyses not involving predicted Ce. Only the surgical period was analyzed, and no conclusions can be made concerning hysteresis during induction or emergence which might occur in states of cognitive dysfunction. Reporting the predicted propofol Ce is better than reporting the dose and helps refine our clinical observations. However, without blood concentrations predicted Ce is subject to bias and inaccuracies which limits the ability to make definitive conclusions concerning pharmacodynamic interactions between cognition and sedation requirements. Although ignoring the variability of individual measurements around the mean level over time during surgery, using the mean levels of Ce, OAA/S and BIS during surgery to study their interrelationship with other variables reflects the common practice of maintaining the sedation around a goal level, whether implicitly or not, in clinical management. Lastly, this study performed secondary analysis using existing trial data, with fixed sample size not specifically powered to confirm any hypotheses tested in this study. Although age was identified as significantly modifying the association between Ce and OAA/S, we were not able confirm similar effect modification by pre-operative cognitive state measured by CDR score or other baseline cognitive variables using the STRIDE data.

## Conclusion

In summary, during sedation under spinal anesthesia, AvgBIS and AvgOAA/S score are good proxies of predicted AvgCe during surgery in routine clinical practices, as evident in the high correlation and 0 systematic bias demonstrated in the results. Furthermore, the relationships between the predicted AvgCe and both AvgBIS and AvgOAA/S score do not appear to be altered by baseline cognitive impairment. None of the baseline cognitive variables studied in STRIDE independently exerted an effect on either the AvgBIS number or AvgOAA/S score.

## Data Availability

The datasets used and/or analyzed during the current study are available from the corresponding author on reasonable request.

## Abbreviations

AD: Alzheimer’s Disease
AvgBIS: Average Bispectral Index
AvgCe: Mean Predicted Ce Level
AvgMAP: Average Mean Arterial Blood Pressure
AvgOAA/S: Average Observer’s Assessment of Alertness/Sedation Scale
B-A: Bland-Altman
BIS: Bispectral Index
CAM: Confusion Assessment Method
CCI: Charlson Comorbidity Index
CDR: Clinical Dementia Rating
Ce: Predicted Propofol Effect Site Concentration
CSF: Cerebrospinal Fluid
DRS-R-98: Delirium Rating Scale-R-98
DST: Digit Span Test
GDS: Geriatric Depression Scale
LOWESS: Locally Weighted Scatterplot Smoothing
MAP: Mean Arterial Blood Pressure
MMSE: Mini-mental Status Exam
OAA/S: Observer’s Assessment of Alertness/Sedation Scale
SD: Standard Deviations
STRIDE: A Strategy to Reduce the Incidence of Postoperative Delirium in Elderly Patients
